# DIALIGN-T: An improved algorithm for segment-based multiple sequence alignment

**DOI:** 10.1186/1471-2105-6-66

**Published:** 2005-03-22

**Authors:** Amarendran R Subramanian, Jan Weyer-Menkhoff, Michael Kaufmann, Burkhard Morgenstern

**Affiliations:** 1 University of Tübingen, Wilhelm-Schickard-Institut für Informatik, Sand 13, 72076 Tübingen, Germany; 2 University of Göttingen, Institute of Microbiology and Genetics, Goldschmidtstr. 1, 37077 Göttingen, Germany

## Abstract

**Background:**

We present a complete re-implementation of the segment-based approach to multiple protein alignment that contains a number of improvements compared to the previous version 2.2 of *DIALIGN*. This previous version is superior to Needleman-Wunsch-based multi-alignment programs on *locally *related sequence sets. However, it is often outperformed by these methods on data sets with *global *but weak similarity at the primary-sequence level.

**Results:**

In the present paper, we discuss strengths and weaknesses of DIALIGN in view of the underlying *objective function*. Based on these results, we propose several heuristics to improve the segment-based alignment approach. For pairwise alignment, we implemented a fragment-chaining algorithm that favours chains of low-scoring local alignments over isolated high-scoring fragments. For multiple alignment, we use an improved *greedy *procedure that is less sensitive to spurious local sequence similarities. To evaluate our method on globally related protein families, we used the well-known database *BAliBASE*. For benchmarking tests on locally related sequences, we created a new reference database called *IRMBASE *which consists of simulated conserved motifs implanted into non-related random sequences.

**Conclusion:**

On BAliBASE, our new program performs significantly better than the previous version of DIALIGN and is comparable to the standard global aligner CLUSTAL W, though it is outperformed by some newly developed programs that focus on global alignment. On the locally related test sets in IRMBASE, our method outperforms all other programs that we evaluated.

## Background

Traditional approaches to multiple sequence alignment are either *global *or *local *methods. Global methods align sequences from the beginning to the end [4,24,9]. Based on the Needleman-Wunsch objective function [18], these algorithms define the *score *of an alignment by adding up scores of *individual *residue pairs and by imposing *gap penalties*; they try to find an alignment with maximum total score in the sense of this definition. By contrast, most local methods try to find one or several conserved motifs shared by *all *of the input sequences [29,12,5].

During the last years, a number of hybrid methods have been developed that combine global and local alignment features [17,19,2,8]. One of these methods is the *segment-based *approach to multiple alignment [17] where alignments are composed from pairwise local sequence similarities. Altogether, these similarities may cover the entire input sequences – in which case a global alignment is produced – but they may as well be restricted to local motifs if no global homology is detectable. Thus, this approach can return global or local alignments – or a combination of both – depending on the extent of similarity among the input sequences.

Instead of comparing single residue pairs, the segment-based approach compares entire *substrings *of the input sequences to each other. The basic building-blocks for pairwise and multiple alignment are un-gapped pairwise local alignments involving two of the sequences under consideration. Such local alignments are called *fragment alignments *or *fragments; *they may have any length up to a certain maximum length *M*. Thus, a fragment *f *corresponds to a *pair of equal-length substrings *of two of the input sequences. Pair-wise or multiple alignments are composed of such fragments; the algorithm constructs a suitable collection *A *of fragments that is *consistent *in the sense that all fragments from *A *can be represented *simultaneously *in one output multiple alignment.

Note that, since multiple alignments are composed of local *pairwise *alignments, conserved motifs are not required to involve *all *of the input sequences. Unlike standard algorithms for local multiple alignment, the segment-based approach is therefore able to detect homologies shared by only two of the aligned sequences. With its capability to deal with both, globally and locally related sequence sets and with its ability to detect local similarities involving only a *subset *of the input sequences, the segment approach is far more flexible than standard methods for multiple alignment. It can be applied to sequence families that are not alignable by those standard methods; this is the main advantage of segment-based alignment compared to more traditional alignment algorithms. The previous implementation of the segment-based multi-alignment approach is DIALIGN 2.2 [16].

During recent years, systematic studies have been carried out on real and artificial benchmark data sets to evaluate the accuracy of multi-alignment programs [26,11,20]. These studies concluded that DIALIGN is superior to other programs if sequence sets with *local *homologlis are to be aligned. On sequences with weak but *global *homology, however, the previous implementation of the program is often out-performed by purely global methods such as CLUSTAL W [24], by hybrid medthods like T-COFFEE [19] or POA [13], or by the recently developed programs MUSCLE [8] and PROBCONS [6] that are currently the best-performing methods for global multiple protein alignment. In the next section, we show that the inferiority of DIALIGN 2.2 on weakly but globally related sequence sets is due to the objective function used by the program. If the program can choose between (a) a *global *pairwise alignment consisting of many fragments with low individual fragment scores and (6) an alternative *local *alignment consisting of only a few isolated fragments with higher individual scores, it tends to prefer the second type of alignment over the first one. Consequently, for sequences with weak but global similarity, DIALIGN is vulnerable to spurious random-similarities.

In this paper, we describe a complete re-implementation of the DIALIGN algorithm that overcomes some of the shortcomings of the previous program version 2.2. The paper is organised as follows: in the next section, we discuss the *objective function *that DIALIGN uses to assess the quality of different alignments for a given input data set. We show that this objective function systematically *over*-estimates isolated local alignments compared with alternative alignments that would extend over the entire length of the sequences. Next, we introduce two heuristics for pairwise and multiple alignment, respectively, to counter-balance this bias towards isolated local similarities. Then we describe additional features of our new implementation, and in in the section *Results and discussion*, we evaluate our software tool and compare it to the previous implementation of DIALIGN and to other standard multi-alignment programs.

## Objective functions for sequence alignment

From a computer scientist's point-of-view, sequence alignment is an *optimisation problem*. Most alignment algorithms are – explicitly or implicitly – based on an *objective function*, i.e. on some kind of *scoring scheme *assigning a quality score to every possible alignment of a given input sequence set. Based on such a scoring scheme, different optimisation algorithms are used to find optimal or near-optimal alignments. For multiple alignment, a variety of optimisation techniques have been proposed. These algorithms differ substantially from each other in view of their computational complexity and in view of their ability to find or approximate numerically optimal alignments. However, the most important feature of an alignment program is not the optimisation algorithm that it uses, but rather the underlying objective function that is used to score possible output alignments. If the objective function is *biologically wrong *by assigning high scores to biologically meaningless alignments, then even the most efficient optimisation algorithms are only efficient in finding mathematically high-scoring *nonsense *alignments. With a more realistic objective function, however, even simple-minded heuristics may lead to biologically plausible alignments.

The objective function that we use in the segment-based approach is defined as follows: each possible fragment (segment pair) *f *is assigned a *weight score w*(*f*) depending on the probability *P*(*f*) of *random occurrence *of such a fragment. More precisely, the program uses a similarity function *s *assigning a score *s*(*a, b*) to each possible pair (*a*, *b*) of residues. For protein alignment, one of the usual substitution matrices can be used; for alignment of DNA or RNA sequences, the program simply distingues between matches and mismatches. For a fragment *f*, its *Needleman-Wunsch score NW *[*f*] is calculated which is defined as the sum of similarity values of aligned nucleotides or amino acid residues (note again, that fragments do not contain gaps). To define the weight score *w*(*f*) of *f*, we consider the probability *P*(*f*) of finding a fragment *f*' of the same length as *f *and with a Needleman-Wunsch score *NW *[*f*'] ≥ *NW *[*f*] in *random sequences *of the same length as the input sequences. *w*(*f*) is then defined as the *negative logarithm *of this probability; see [14] for more details. The *total *score of a – pairwise or multiple – alignment is defined as the *sum of weight scores *of the fragments it is composed of; gaps are not penalised. The idea is that the *less *likely a given fragment collection is to occur just by chance, the *more *likely it is to be biologically relevant so the higher its score should be. Thus, while standard alignment approaches try to find an alignment that is *most likely *under the assumption that the input sequences are related by common ancestry [7], we try to find an alignment that is *most unlikely *under the assumption that the sequences are *not *related. A pairwise alignment in the sense of the above definition corresponds to a *chain *of fragments, and an alignment with maximum total weight score can be found using a recursive fragment-chaining procedure [15]; for multiple alignment, a *greedy *heuristics is used [1,14].

As explained above, DIALIGN defines the score *S*(*A*)of an alignment *A *= {*f*_1_,..., *f*_*k*_} as the sum of weight scores *w*(*f*_*i*_) of its constituent fragments, and these weight scores are, in turn, defined as negative logarithms of probabilities *P*(*f*_*i*_) of their random occurrence. Thus, the score *S*(*A*)is calculated as



and searching for an alignment with *maximal *score is equivalent to searching for a consistent collection of fragments *A *= {*f*_1_,..., *f*_*k*_} with *minimal *product of probabilities ∏_*f*∈*A *_*P*(*f*). But considering the *product *of fragment probabilities means to consider the probability of their *joint *occurrence under the assumption that these events are *independent *of each other. This would be reasonable if we would search for an *arbitrary *fragment collection with low probability of random occurrence. In our approach, however, we require a fragment collection to be *consistent*, so the set of allowed combinations of fragments is drastically reduced. The probability of finding a *consistent *set of fragments is consequently far smaller than the product of the probabilities of finding all of the corresponding *individual *fragments. Thus, by using the product ∏_*f*∈*A *_*P*(*f*), DIALIGN generally *over-estimates *the probability *P*(*A*) of random occurrence of an alignment *A*.

In our context, the crucial point is that the probabilities *P*(*A*) – and therefore the scores *S*(*A*) – are not *uniformly *over-estimated – or under-estimated, respectively – for all possible alignments, but there is wide difference between global and local alignments. For a *global *alignment *A*_*g *_that covers most of the sequences, the *discrepancy *between the *real *probability *P*(*A*_*g*_) of its random occurrence and the approximation *P*(*f*) used by DIALIGN is far more significant than for a *local *alignment *A*_*l*_. This is because a global alignment corresponds to a *dense *collection of fragment, so here the consistency constraints are much tighter than in a local alignment consisting of only a few isolated fragments. As a result, DIALIGN *relatively *over-estimates the probability *P*(*A*_*g*_) of a global aligment *A*_*g *_compared with an alternative local alignment *A*_*l*_, so it *under-estimates *the score *S*(*A*_*g*_) compared with the score *S*(*A*_*l*_).

## Reducing the influence of isolated local similarities

In the previous section, we explained why the objective function used in DIALIGN systematically prefers local alignments over alternative global alignments of the same data set. An improved objective function that would use a better approximation to the probability *P*(*A*)of random occurrence of an alignment *A *would have to take into account the combinatorial constraints given by our consistency condition. Defining such an objective function would be mathematically challenging. For our new program, we therefore use the objective function that has been used in previous versions of DIALIGN. However, we introduce two heuristics to counterbalance the bias in this objective function towards isolated local alignments.

### Excluding low-scoring sub-fragments

The pairwise alignment algorithm that we are using is a modification of the space-efficient fragment-chaining algorithm described in [15]. At each position (*i, j*) in the comparison matrix, this algorithm considers all fragments (= segment pairs) starting at (*i, j*) up to a certain maximum length *M*. For protein alignment, the previous program DIALIGN 2.2 uses a default value of *M *= 40; *M *can be reduced to speed-up the program, but this may result in decreased alignment quality. Initially, the length limitation for fragments has been introduced to reduce the program running time; this way the time complexity of the pairwise fragment-chaining algorithm is reduced from *O*(*l*^3^) to *O*(*l*^2^) where *l *is the maximum length of the two sequences. One might think that increasing the maximum fragment length *M *would result in improved alignment quality. In fact, we observed that with slightly increased values for *M*, better alignments were obtained, but with values *M *> 50, the quality of the produced alignments decreased dramatically.

In systematic test runs, we observed that for large values of *M*, output alignments often contain long fragments involving a mixture of high-scoring and low-scoring sub-fragments. With an ideal objective function, a single long fragment *f *containing low-scoring sub-fragments would automatically receive a *lower *score than the chain of short fragments that would be obtained from *f *by removing those low-scoring sub-fragments. As a result, output alignments would tend to consist of shorter fragments rather than of longer fragments with low-scoring sub-regions. For reasons explained in the previous section, however, the scoring scheme used by DIALIGN over-estimates single long fragments compared with chains of smaller fragments that would be obtained by removing low-scoring regions from those long fragments.

In our new approach, we use the following heuristics to prevent the algorithm from selecting long fragments with low-scoring sub-regions. We define a length threshold *L *for low-quality sub-fragments. Sub-fragments of length ≥ *L *with negative Needleman-Wunsch score are allowed within short fragments but are excluded in fragments of length ≥ *T *where *T *<*M *is a parameter that can be adjusted bu the user. For a pair of input sequences *S*_1 _and *S*_2 _and given values for the parameters *T*, *M *and *L*, our new algorithm proceeds as follows. Let *f*(*i, j, k*) denote the fragment of length *k *that starts at position *i *in sequence *S*_1_and at position *j *in sequence *S*_2_, respectively. By *S*_1 _[*k*], we denote the *k*-th character in sequence *S*_*i*_. As in the original DIALIGN algorithm, we traverse the comparison matrix for *S*_1 _and *S*_2_, and at every position (*i, j*), we consider fragments *starting *at this position; suitable fragments are then added to a growing set *F *of candidate fragments from which the algorithm selects a fragment chain with maximum total score with respect to the underlying objective function [15]. If a region of low quality occurs, the maximum fragment length *M*(*i, j*) for fragments starting at (*i, j*) is reduced from *M *to *T*. More formally, we perform the following steps for fragments starting at a fixed position (*i, j*):

1. Initially, the maximum length for fragments starting at (*i, j*) is *M*(*i, j*) = *M*.

2. We start with length *k *= 1, i.e. we consider the fragment *f*(*i, j*, 1).

3. If the current fragment length *k *exceeds *M*(*i, j*) then the procedure stops and we continue with fragments starting at (*i, j *+ 1).

4. If the similarity score *s*(*S*_1 _[*i *+ *k *- 1], *S*_2 _[*j *+ *k *- 1]) of the last residue pair in *f*(*i, j, k*) is not negative, we take the fragment *f*(*i, j, k*) into account by adding it to the set *F *and continue with step 7. Otherwise we detected the potential beginning of a low-quality sub-fragment starting at positions *i *+ *k *- 1 and *j *+ *k *- 1, respectively.

5. In this case we do a lookahead and calculate the NW-score of the potential low-scoring fragment *f*(*i *+ *k *- 1, *j *+ *k *- 1, *L*) which is defined as



6. If *NW *[*f*(*i *+ *k *- 1, *j *+ *k *- 1, *L*)] < 0, we actually detected a low-quality sub-fragment. If *k *>*T*, the procedure stops and no further increasing of *k *is being considered, otherwise we set *M*(*i, j*) = *T*.

7. The length *k *is incremented by 1 and we continue with the step 3.

By default, our program uses a length threshold for low-quality sub-fragments of *L *= 4 and the maximum length of fragments containing such regions of low quality is *T *= 40. These values have been determined based on systematic test runs on BAliBASE. At this point, we want to mention the impact of the parameters *L *and *T *on the quality of the produced output alignments. For example, with values *L *= 3 or *L *= 5 the alignment quality is dramatically worsened compared with the default value *L *= 4.

Our stop criterion for low-scoring sub-fragments not only improves the quality of the resulting alignments but also reduces the program running time. The runtime of our pairwise algorithm is proportional to the number of fragments that are considered for alignment. Thus, the *worst-case *time complexity is *O*(*l*_1_·*l*_2_·*M*) where *l*_1 _and *l*_2 _are the lengths of the input sequences. By excluding long fragments with low-scoring sub-fragments, we ignore a large number of fragments that would have been considered for alignment in previous program versions. Therefore, our new heuristics allows us to *increase *the maximum possible fragment length from *M *= 40 to *M *= 100 without excessively increasing the total number of fragments that are to be looked at. A further extension of *M *is prohibited due to numerical instabilities. Altogether, the resulting alignments can reflect the extension of existing homologies more realistically than the previous version of DIALIGN with only a moderate increase in program running time.

### Weight score factors

As mentioned above, DIALIGN uses a greedy optimisation procedure for multiple alignment. The order in which fragments are included into the multiple alignment is determined based on their *weight scores*. A general problem with this greedy approach is that if a wrong fragment is accepted for multiple alignment, it cannot be removed later on. Note that even a single wrong choice in the greedy procedure can impair the quality of the resulting alignment dramatically. Thus, special care has to be taken to prioritise fragments for the greedy algorithm. We observed that in many cases spurious but high scoring fragments from pairwise alignments are *inconsistent *with a good overall multiple alignment. Due to their weight scores, however, such fragments may be incorporated into the multiple alignment by the original DIALIGN, thereby leading to output alignments of lower quality.

As explained in the previous section, the weight score of a fragment depends on the probability of its *random occurrence *in sequences the length of the input sequences. Thus, weight scores are purely based on *intrinsic *properties of fragments – and on the length of the input sequences – but they do not take into account the *context *of a fragment within the pairwise alignment. In reality, however, the context of a fragment is crucial to assess its reliability. If a fragment *f *is part of a high-scoring pairwise alignment, then *f *is, of course, far more likely to be biologically significant than if the same fragment *f *would be found isolated in otherwise un-related sequences. Therefore the overall similarity among two sequences should be taken into account if fragments are ranked prior to the greedy procedure.

In our new program, we adopt the following approach: we multiply the weight score of each fragment by the square of the total weight score of the respective sequence pair divided by the overall weight score of all pairwise alignments. Let *S*_1_,..., *S*_*n *_be the input sequences and let *f *be a fragment involving sequences *S*_*i *_and *S*_*j*_. Next, let *w*(*S*_*i*_, *S*_*j*_) denote the total weight score of the pairwise alignment for *S*_*i *_and *S*_*j *_– i.e. the sum of weight scores of an optimal chain of fragments – and let *W *be the total sum of weight scores of all pairwise alignments. That is, we define



We then define the *adjusted *weight score



and in our greedy algorithm, fragments are sorted according to their adjusted scores *w*'(*f*). This way, we prefer fragments belonging to sequence pairs of high similarity over those from weakly related sequence pairs. Altogether, this weight adjustment respects the similarity of the sequence pairs better than the previous method and hence may keep the greedy procedure from adding isolated spurious fragments that would have led to a lower-scoring and biologically less meaningful output alignment. The sorted list of fragments from the optimal pairwise alignments are kept in a binary heap structure that can be updated efficiently when inconsistent fragments are to be removed or modified as explained in the next section.

## Further program features

### Dealing with inconsistent fragments

In the original DIALIGN approach, an inconsistent fragment *f *is *completely *discarded in the greedy procedure – even if just a few residue pairs are inconsistent with the current alignment. In such a situation, it would be of course more sensible to remove only those inconsistent residue pairs from *f *and to give the remaining sub-fragments a second chance in the greedy selection process. It is easy to see that a fragment *f *is consistent with an existing alignment *A *if and only if each *pair *of aligned residues in *f *is consistent with *A*. In our new implementation, we use the following procedure for non-consistent fragments. An inconsistent fragment *f *is processed from left to right. Starting with the left-most residue pair, we remove all inconsistent residue pairs until we find the first consistent pair *p*. Next, we consider all consistent residue pairs starting with *p *until we find again an inconsistent residue pair. This way, we obtain a consistent sub-fragment *f*' of *f *for which we calculate the weight score *w*(*f*'). By construction, *f*' is consistent with the existing alignment and could, in principle, be added to the list of accepted fragments.

However, we do not immediately include *f*' into the growing multiple alignment since the score *w*(*f*') might be smaller than the original score *w*(*f*). Instead, we insert *f*' at the appropriate position in our sorted list of fragments depending on its adjusted weight score *w*'(*f*'). With the binary heap structure mentioned in the previous section, consistent sub-fragments of inconsistent fragments can be efficiently re-positioned according to their newly calculated adjusted weights. The remainder of *f *is treated accordingly, i.e. inconsistent residue pairs are removed and the remaining consistent sub-fragments are inserted at appropriate positions in the list of candidate fragments. Note that with our weighting function *w*, the weight score *w*(*f*') of a sub-fragment *f*' contained in a fragment *f *can in general be *larger *than the weight *w*(*f*). In the above situation, however, we have necessarily *w*(*f*') ≤ *w*(*f*') [and therefore *w*'(*f*') ≤ *w*'(*f*)] since we assumed that *f *is part of the optimal pairwise alignment of two sequences. If the score *w*(*f*') of a sub-fragment of *f *exceeded *w*(*f*), then *f*' would have been selected for the optimal pairwise alignment instead of *f*.

### Probability estimates

The previous implementation DIALIGN 2.2 uses pre-calculated probability tables to calculate fragment weight scores; these tables are based on the BLOSUM 62 substitution matrix. They have been calculated years ago and are difficult to re-calculate if a user wants to employ another similarity matrix. It is therefore not possible to run DIALIGN 2.2 with substitution matrices other than BLOSUM 62. In our new implementation, we use a rather efficient way to estimate the probabilities that are used for our weight score calculations. We pre-calculated probability tables for a variety of substitution matrices. In addition, the user can re-calculate these tables 'on the fly' for arbitrary matrices with a moderate increase in program running time.

As explained in section *Objective functions for sequence alignment*, we define the weight score of a fragment *f *involving sequences *S*_*i *_and *S*_*j *_as

*w*(*f*) = - *logP*(*f*)

where *P*(*f*) denotes the the probability for the occurence of a fragment *f*' of the same length as *f *and with Needleman-Wunsch score *NW *[*f*'] ≥ *NW *[*f*] in *random sequences *of the same length as *S*_*i *_and *S*_*j*_. By *random sequences *we mean *independent identically distributed *(*iid*) sequences where each residue occurs at any position with probability 1/4 for nucleic acid sequences and 1/20 for protein sequences, respectively. In the following, we outline how our program approximates the probabilities *P*(*f*).

In a first step, we estimate the probability  of finding a fragment *f*' of length *n *and with Needleman-Wunsch score *NW *[*f*'] ≥ *s *in random sequences of length 2·*n*. Note that  depends on the underlying substitution matrix but not on the length or composition of the input sequences *S*_*i *_and *S*_*j*_. The numerical values  are estimated as follows:

1. Random experiments are performed to obtain a preliminary estimates  for . The experimental values  should approximate  with sufficient accuracy for values of *n *and *s *where enough experimental data are avail able. This is the case if  is not too small.

2. For small values of , we first compute the probabilitys *P*_1_(*s, n*) for a *single *random fragment *f*' of length *n *to have a Needleman-Wunsch score *NW *[*f*'] ≥ *s*. *P*_1_(*s, n*) can be easily calculated as a sum of convolution products. Similar as in [14], small values of  are estimated using the approximation formula



3. All in all, we define  for a given value s by first considering the trivial case *n *= 1 and then defining for *n *= 2,..., *M*:



The described procedure to estimate  is computationally demanding. Since the values  do not depend on the input sequences, we *pre-calculated *these probabilities for several standard substitution matrices and stored their values in auxiliary files from where they are retrieved during the program run.

In a second step, we use  to estimate the probability *P*(*s, n*) for finding a fragment *f*' of length *n *with Needleman-Wunsch score *NW *[*f*'] ≥ *s *in sequences the length of the input sequences. This step is computationally less expensive and can therefore be carried out during the program run. Let *l*_*i *_and *l*_*j *_be the lengths of the input sequences *Si *and *S*_*j*_, respectively. Similar as in [14], we compute *P*(*s, n*) as



where *P*_*T *_is a threshold parameter. During a program run, the values *P*(*s, n*) are calculated for all possible values of *n *and *s before *the pairwise alignment of sequences *S*_*i *_and *S*_*j *_is carried out. The negative logarithms -log *P*(*s, n*) are stored in a look-up table where they are retrieved during the pairwise alignment to define the fragment scores.

We pre-calculated the probabilities  for several substitution matrices of the BLOSUM family. To determine the experimental probability values *P*_*exp*_(*s, n*), we carried out 10^6 ^random experiments for each relevant pair of parameters (*s, n*). Here, we considered values for *n *betwen 1 and a maximum fragment length *M *= 100. Files with the resulting values of  values are distributed together with our software package. To calculate *P*(*f*), we use a threshold probability *P*_*T *_= 10^-8^. Our program can also be used to calculate the values  for *arbitrary *user-defined substitution matrices. Calculating these values using 10^5 ^random experiments for each value of *n *and *s *takes around 20 minutes on a Linux workstation (RedHat 8.0) with an 1.5 Ghz Pentium 4 processor and 512 MB Ram. In our experience, 10^5 ^random experiments are sufficient to obtain high-quality probability estimates.

## Results and discussion

We evaluated the performance of our program and compared it to alternative multi-alignment software tools using a wide variety of benchmark sequences. As a first set of reference data, we used the well-known BAliBASE 2.1 [25]. BAliBASE has been used in numerous studies to test the accuracy of multiple-protein-alignment software. It should be mentioned that, although some of the reference sequences in BAliBASE contain insertions and deletions of moderate size, BAliBASE is heavily biased towards *globally *related protein families. All BAliBASE sequences contain homologous *core blocks *with verified 3D structure; alignment programs are evaluated according to their ability to correctly align these blocks. According to the BAliBASE authors, these core blocks cover 58 % of the residues in the database. However, sequence similarity is clearly not restricted to those regions of verified 3D structure so, in reality, far more than 58 % of the total sequence length are homologous to other sequences in the respective sequence families. Also, the sequences in BAliBASE are not realistic full-length sequences, but they have been truncated by the BAliBASE developers in order to remove non-related parts of the sequences. As a result, BAliBASE consists almost entirely of *globally *related sequence sets; this is why *global *alignment programs such as CLUSTAL W perform best on these benchmark data.

To study the performance of alignment programs on *locally *related sequence sets, Lassmann and Sonnhammer used artificial random sequences with implanted conserved motifs [11]. Random sequences are frequently used to evaluate computational sequence analysis tools; they are particularly useful to study the *specificity *of a tool, see e.g. [23,10,20]. Unfortunately, the benchmark data by Lassmann and Sonnhammer are not publicly available. Therefore, we set up our own benchmark database for local multiple protein alignment that we called *IRMBASE *(Implanted Rose Motifs Base).

As Lassmann and Sonnhammer did in their previous study, we produced groups of artificial conserved sequence motifs using the ROSE software tool [23]. ROSE simulates the process of molecular evolution. A set of 'phylogenetically' related sequences is created from a user-defined 'ancestor' sequence according to a phylogenetic tree. During this process sequence characters are randomly inserted, deleted and substituted under a pre-defined stochastic model. This way, a sequence family with known 'evolution' is obtained, so the 'correct' multiple alignment of these sequences is known. Note that these alignments contain mismatches as well as gaps. We inserted families of conserved motifs created by ROSE at randomly chosen positions into non-related *random *sequences. This way, we produced three reference sets *ref1, ref2 *and *ref3*, of artificial protein sequences. Sequences from ref1 contain one motif each and sequences from ref2 and ref3 contain two and three motifs each, respectively. Each reference set consists of 60 sequence families, 30 of which contain ROSE motifs of length 30 while the remaining 30 families contain motifs of length 60. 20 sequence families in each of the reference sets consist of 4 sequences each, another 20 families consist of 8 sequences while the remaining 20 families consist of 16 sequences. In ref1, random sequences of length 400 are added to the conserved ROSE motif while for ref2 and ref3, random seqences of length 500 are added.

For both BAliBASE and IRMBASE, we used two different criteria to evaluate multi-alignment software tools. We used the *sum-of-pair score *where the percentage of correctly aligned *pairs *of residues is taken as a quality measure for alignments. In addition, we used the *column score *where the percentage of correct *columns *in an alignment is the criterion for alignment quality. Both scoring schemes were restricted to *core blocks *within the reference sequences where the 'true' alignment is known. For IRMBASE, the core blocks are defined as the conserved ROSE motifs. In general, the sum-of-pairs score is more appropriate than the column score because this latter score ignores all correctly aligned residues in an alignment column if one single residue in this column is mis-aligned. However, there are situations where the column score is more meaningful than the sum-of-pairs score. This is the case, for example, for BAliBASE reference sets containing 'orphan sequences'.

To compare the output of different programs to the respective benchmark alignments, we used C. Notredame's program aln_compare [19]. Tables 1 and 2 summarise the performance of DIALIGN-T, DIALIGN 2.2, CLUSTAL W, MUSCLE, PROBCONS, T-COFFEE and POA on IRMBASE while Tables 3 and 4 show their accuracy on BAliBASE. In addition, Tables 5, 6, 7 and 8 contain the percentage of sequence sets where DIALIGN-T is outperformed by the other programs that we tested. Tables 1 and 2 show that, on locally related sequence families, DIALIGN-T is significantly superior to the algorithms DIALIGN 2.2, T-COFEE, MUSCLE, POA and CLUSTAL W. Only DIALIGN-T, DIALIGN 2.2, T-COFFEE and (in a very reduced way) PROBCONS produced reasonable results on IRMBASE 1.0. However, DIALIGN-T is the fastest and most accurate amongst all methods that we looked at. We would like to emphasize that the performance of multi-alignment methods on *simulated *data only roughly reflects their performance on *real *data. Nevertheless, in the absence of real-world benchmark data for local multiple alignment, the results on IRMBASE can give us an idea of how different algorithms deal with locally conserved motifs.

For globally related sequence families, Tables 3 and 4 show that, on average, DIALIGN-T outperforms DIALIGN 2.2 and POA on BAliBASE 2.1 while its performance is similar to CLUSTAL W. By contrast, the previous version DIALIGN 2.2 is clearly outperformed by CLUSTAL W on these data sets. Finally, DIALIGN-T is still outperformed on many of the BAliBASE test sequences by T-COFFEE, MUSCLE and PROBCONS; the latter program is currently the best-performing multiple aligner on BAliBASE. The superiority of our new approach compared to DIALIGN 2.2 and POA is clearly *statistically significant *according to the Wilcoxon Matched Pairs Signed Rank Test. On BAliBASE reference sets ref1, ref2 and ref3 where sequences contain only small insertions and deletions, the performance of DIALIGN-T is roughly comparable to CLUSTAL W, but yet still significantly worse than T-COFFEE, PROBCONS or MUSCLE. Our program is statistically significantly superior or equal to all tested methods, except MUSCLE and PROBCONS, on the sequence sets with larger insertions or deletions (ref4 and ref5 of BAliBASE).

Overall, the *relative *performance of the different alignment tools is similar under the two alternative evaluation criteria that we used (sum of pairs and column score) – although, the *absolute *values of the column scores are, of course, lower than the sum-of-pairs scores. Maybe surprisingly, both versions of DIALIGN are superior to all other programs in our study on the locally related sequences from IRMBASE – while on the other hand, DIALIGN was outperformed by alternative methods on reference sets 4 and 5 of BAliBASE. These sequence sets are also considered locally related because they contain larger insertions and deletions then other BAliBASE sequences. The reason for this apparent discrepancy is that the ref4 and ref5 sequence sets in BAliBASE are not truly locally related, but they still show some similarity outside the conserved core blocks. In IRMBASE, by contrast, sequence similarity is strictly limited to the conserved motifs.

Since we re-implemented the DIALIGN algorithm *from scratch *and used a variety of novel program features, it is not possible to tell exactly to what extend each of these features contributed to the improved program performance. Systematic test runs with varying parameters indicate, however, that the superiority of our new program compared to the previous program DIALGIN 2.2 on locally as well as on globaly related sequence families is mainly due to the program features explained in the third section. The improvement that we achieved with these heuristics is statistically significant. The features explained in section *Further program features *also improved the program accuracy, though here the improvement was not statistically significant.

Table 9 shows the program running time for the seven programs that we tested in our study. DIALIGN-T is around 6 % slower than the previous implementation DIALIGN 2.2 on BAliBASE 2.1, but on IRMBASE, DIALIGN-T is approximately 30 % faster than DIALIGN 2.2. In DIALIGN, the CPU time for multiple alignment is mainly spent on *pairwise *alignments that are performed before fragments are included into the multiple alignment. As explained in section *Excluding low-scoring sub-fragments*, the runtime for pairwise alignment is roughly proportional to the number of fragments that are considered for alignment and, for sequences of length *l*_1 _and *l*_2 _and a maximum fragment length *M*, up to *l*_1 _× *l*_2 _× *M *fragments are to be considered. In our new program DIALIGN-T, the maximum fragment length *M *is increased to 100 compared to 40 for the original DIALIGN program. Nevertheless, the program running time is only slightly increased for the globally related protein families from BAliBASE and considerably *decreased *for the locally conserved sequences from IRMBASE. This is due to the heuristic *stop criterion *for fragments introduced above. The slowest program in our comparison was T-COFFEE which is more than eleven times slower than DIALIGN-T on IRMBASE and more than five times slower on BAliBASE. POA was the fastest method. On BAliBASE, the program PROBCONS produces the best results in terms of alignment accuracy. The program is, however, the second slowest program after T-COFFEE on both BAliBASE and IRMBASE. MUSCLE provides so far the best tradeoff between running time and quality on globally related sequence families, but when it comes to local alignments both running time and alignment quality decrease drastically. The memory consumption of our method has been improved compared to DIALIGN 2.2.

With the development of DIALIGN-T, we significantly improved the segment-based approach to multiple protein alignment on both local and global benchmark data. The new heuristics that we introduced, generally favour consistent groups of low-scoring fragments over isolated higher-scoring fragments. This way, we improved the program performance on globally related sequence sets where the segment approach was previously inferior to programs such as CLUSTAL W and POA. On these data sets, our new method is significantly more accurate but only slightly slower than DIALIGN 2.2. On BAliBASE, the performance of our approach is now comparable to the popular global alignment program CLUSTAL W. For locally related protein families, DIALIGN-T performs significantly better and is also considerably faster than the previous DIALIGN 2.2 which was, so far, the best available method on locally related protein families. In addition to these improvements, it is now possible to use arbitrary user-defined substitution matrices which was not possible for the original DIALIGN program. To further enhance the performance of our method, we are planning to improve the greedy algorithm that DIALIGN uses for multiple alignment. Rather than focusing on *pairwise *fragment alignments, we will develop heuristics that are able to integrate *multiple *local alignments into the final multiple alignment. This approach should further improve the sensitivity of our methods for locally conserved motifs.

Finally, we would like to make some general remarks on parameter tuning and program evaluation in multiple sequence alignment. As mentioned above, we identified suitable values for our parameters *T *and *L *based on test runs with BAliBASE, and we assume that this is how the program parameters for most multiple protein aligners have been tuned during the last years. Therefore, the question has been raised if current protein alignment programs are *overfitted *with respect to BAliBASE. Parameter overfitting is a serious problem for many Bioinformatics algorithms. For example, many gene-prediction programs have a large number of parameters to adjust, so it is easy to tune these programs to perform well on a given set of training data. For such programs it is therefore absolutely necessary to clearly separate training data that are used for parameter tuning from test data that are used to evaluate the program. The situation is totally different in multiple alignment. Most multi aligners have only a very small number of parameters to adjust. For our algorithm, for example, the only important parameters to tune are *T *and *L*. BAliBASE, on the other hand, comprises a large variety of test sequences for global multiple alignment. It consists of 139 sequence sets, each of which contains several core blocks, so there is a total of several hundred core blocks that are used to test alignment quality. It is absolutely impossible to tune a small number of parameters in such a way that they work well only on BAliBASE but not on other globally related protein sequences. Thus, if an alignment program performs well on BAliBASE, one can safely assume that it also works well on other globally related protein sequences, even if BAliBASE has been used to adjust its parameter values. In fact, it turned out that the parameters that we tuned on BAliBASE work not only well for these *global *test data but also on the totally different artificial *local *test sequences from IRMBASE.

The real problem with BAliBASE is its heavy bias towards *globally *related sequence sets. This does not only refer to the selection of protein families that are included into BAliBASE. As mentioned above, many protein sequences in the current release of BAliBASE are *not *real-world protein sequences, but have been *artificially truncated *by the developers of BAliBASE in order to *make *them globally related. With these *non-realistic *global test sequences, the BAliBASE authors carried out a systematic program evaluation and – not surprisingly – found out that *Global alignment programs generally performed better than local methods *[26]. The picture could have been totally different if realistic full-length proteins had been used instead of truncated sequences. To counterbalance the bias towards global test sets in BAliBASE, we created an additional benchmark data set consisting of simulated conserved domains embedded in non-related random sequences. The performance of alignment programs on artificial sequences should not be over-estimated as the design of such data sets is necessarily somewhat arbitrary. Nevertheless, our test runs on these simulated data give a rough impression of how different alignment methods perform on locally related data sets. Further systematic studies should be carried out to evaluate the performance of multiple-protein aligners under varying conditions using, for example, the full-length BAliBASE sequences or newly developed benchmark databases such as SABmark [27,28], Prefab [8] or Oxbench [21].

As a concluding remark, we would like to address a fundamental limitation of most multi-alignment methods, including the one presented in this paper: these methods implicitly assume that homologies and conserved motifs occur in the same *relative order *within the input sequences. There are two major reasons for making this assumption. First, an order-preserving multiple alignment that represents homologies by inserting gap characters into the input sequences provides a convenient visualisation of existing homologies. Second – and more importantly -, the order-preservation constraint greatly reduces the *noise *created by random similarities. A program that would return *all *detectable local or global similarities among the input sequences without the above ordering constraints would necessarily return many spurious random similarities. To reduce this noise, arbitrary threshold parameters would have to be applied which, in turn, could prevent a program from detecting some of the *real *homologies. With the ordering constraint that is implicitly imposed by most alignment programs, weak homologies can be detected, provided they are order-consistent with other detected similarities, i.e. if they fit into one single output alignment. Many evolutionary events such as insertions, deletions and substitutions preserve the relative ordering among sequence homologies. In this situation order-respecting alignment methods are, in principle, able to represent all true biological homologies in one multiple alignment. Nevertheless, for distantly related protein families, non-order-preserving events such as duplications or translocations need to be taken into account. Such events play an important role in comparative analysis of *genomic *sequences which became an important area of research during the last years [20]. Recently, some promising algorithms for multiple alignment of genomic sequences have been proposed that are able to deal with non-order-conserving evolutionary events [22,3]. Further progress in multiple protein alignment can be expected if these ideas are applied to protein alignment algorithms.

## Program availability

DIALIGN-T is available at *Göttingen Bioinformatics Compute Server ****(GOB-ICS)***:

• **Project name: **DIALIGN-T

• **Project home page: **

• **Operating system(s): **UNIX and LINUX

• **Programming language: **C

• **Other requirements: **none

• **License: **GNU LGPL

• **Any restrictions to use by non-academics: **none

A program package with functionalities to compute alignments of nucleic acid sequences will be available soon.

## Authors' contributions

ARS conceived the new heuristics, implemented the program, constructed IRMBASE, did most of the evaluation and wrote minor parts of the manuscript; JWM participated in program evaluation; MK provided resources; BM supervised the work, provided resources and wrote most of the manuscript.

## Figures and Tables

**Figure 1:**
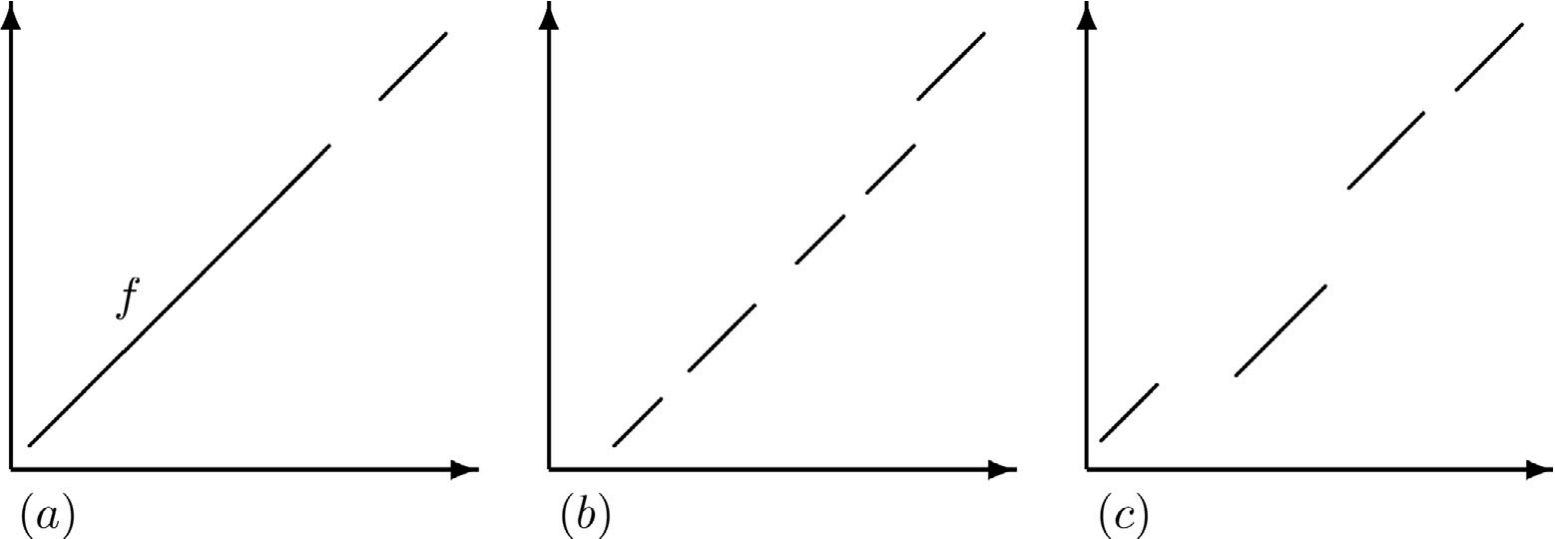
Exclusion of low-scoring regions from alignment fragments. The scoring scheme used in DIALIGN gives relatively high weight scores to single fragments with high Needleman-Wunsch scores (*a*). In our new approach, we exclude low-scoring sub-regions within long fragments by applying a stop criterion for fragment extension. This can result in the replacement of a long fragment *f *by multiple sub-fragments (*b*) or in a completely different alignment (*c*).

**Table 1:** Performance of seven protein multi-alignment programs on the IRMBASE 1.0 database of benchmark alignments. Percentage values are *sum-of-pairs scores*, i.e. the percentage of correctly aligned *residue pairs *of ROSE motifs contained in the IRMBASE sequence families.

Method	ref1	ref2	ref3	Total
DIALIGN-T	94.07%	92.69%	92.68%	93.14%
DIALIGN 2.2	92.26%	92.72%	91.87%	92.28%
T-COFFEE 1.37	91.18%	85.61%	87.81%	88.20%
PROBCONS 1.09	66.74%	68.30%	77.92%	70.98%
POA V2	90.26%	43.61%	36.85%	56.91%
MUSCLE 3.5	36.16%	37.84%	52.30%	42.10%
CLUSTAL W 1.83	8.02%	12.69%	20.16%	13.62%

**Table 2:** Performance of seven protein multi-alignment programs on IRMBASE using *column scores *as quality criterion. Thus, percentage values denote the percentage of correct *alignment columns *of the ROSE motifs in IRMBASE

Method	ref1	ref2	ref3	Total
DIALIGN-T	82.28%	78.36%	79.71%	80.12%
DIALIGN 2.2	79.46%	77.82%	78.24%	78.51%
T-COFFEE 1.37	75.35%	66.60%	69.21%	70.19%
PROBCONS 1.09	33.13%	37.95%	51.26%	40.78%
POA V2	73.00%	12.46%	07.45%	30.97%
MUSCLE 3.5	09.41%	10.89%	22.37%	14.22%
CLUSTAL W 1.83	00.00%	00.83%	05.14%	01.92%

**Table 3:** Performance of seven protein multi-alignment programs on the BAliBASE benchmark database using *sum-of-pairs scores *as evaluation criterion.

Method	ref1	ref2	ref3	ref4	ref5	Total
DIALIGN-T	82.76%	91.28%	75.34%	86.43%	93.30%	84.69%
DIALIGN 2.2	81.40%	89.56%	68.93%	91.24%	94.14%	83.59%
T-COFFEE 1.37	84.67%	93.24%	80.32%	75.80%	96.20%	85.95%
PROBCONS 1.09	90.37%	94.61%	84.34%	89.20%	98.07%	91.11%
POA V2	74.66%	88.32%	63.14%	82.62%	76.71%	76.76%
MUSCLE 3.5	88.25%	93.59%	82.36%	85.62%	97.80%	89.21%
CLUSTAL W 1.83	86.43%	93.22%	75.79%	81.09%	86.10%	86.15%

**Table 4:** Performance of seven protein multi-alignment programs on BAliBASE using *column scores*.

Method	ref1	ref2	ref3	ref4	ref5	Total
DIALIGN-T	73.22%	43.43%	44.69%	66.13%	77.05%	65.65%
DIALIGN 2.2	71.49%	37.42%	35.03%	81.88%	84.47%	64.82%
T-COFFEE 1.37	75.32%	53.44%	52.20%	45.09%	86.96%	68.20%
PROBCONS 1.09	83.21%	59.76%	61.34%	71.09%	91.86%	77.23%
POA V2	63.21%	39.02%	25.57%	57.22%	47.18%	54.18%
MUSCLE 3.5	80.79%	56.37%	56.74%	62.65%	91.57%	74.13%
CLUSTAL W 1.83	78.39%	56.24%	48.87%	50.44%	63.89%	68.48%

**Table 5:** Percentage of sequence families where DIALIGN-T is outperformed on IRMBASE 1.0 by alternative methods according to the *sum-of-pairs score*. The symbol ^+ ^denotes statistically significant superiority, ^- ^statistically significant inferiority and ^0 ^non-significant superiority or inferiority of DIALIGN-T, respectively. Significance has been calculated according to the Wilcoxon Matched Pairs Signed Rank Test with *p *≤ 0.05).

Method	ref1	ref2	ref3	Total
DIALIGN 2.2	20.00%^+^	23.33%^0^	23.33%^+^	22.22%^+^
T-COFFEE 1.37	40.00%^0^	31.67%^+^	41.67%^+^	37.78%^+^
PROBCONS 1.09	20.00%^+^	15.00%^+^	21.67%^+^	18.89%^+^
POA V2	16.67%^+^	0.00%^+^	0.00%^+^	5.55%^+^
MUSCLE 3.5	5.00%^+^	5.00%^+^	0.00%^+^	3.33%^+^
CLUSTAL W 1.83	0.00%^+^	0.00%^0^	0.00%^0^	0.0%^+^

**Table 6:** Percentage of sequence families where DIALIGN-T is outperformed on IRMBASE 1.0 by other methods according to the *column score*. Notation is as in Table 5.

Method	ref1	ref2	ref3	Total
DIALIGN 2.2	11.67%^+^	21.67%^0^	23.33%^+^	18.89%^+^
T-COFFEE 1.37	36.67%^0^	30.00%^+^	26.67%^+^	31.11%^+^
PROBCONS 1.09	18.33%^+^	01.67%^+^	16.67%^+^	16.67%^+^
POA V2	15.00%^+^	00.00%^+^	00.00%^+^	05.00%^+^
MUSCLE 3.5	05.00%^+^	05.00%^+^	00.00%^+^	03.33%^+^
CLUSTAL W 1.83	00.00%^+^	00.00%^+^	00.00%^+^	00.00%^+^

**Table 7:** Percentage of sequence families where DIALIGN-T is outperformed on BAliBASE 2.1 by other methods according to the *sum-of-pairs score*. Notation is as in Table 5.

Method	ref1	ref2	ref3	ref4	ref5	Total
DIALIGN 2.2	28.05%^+^	21.74%^+^	16.67%^+^	16.67%^0^	41.67%^0^	26.24%^+^
T-COFFEE 1.37	58.54%^-^	86.96%^-^	75.00%^-^	25.00%^0^	50.00%^0^	60.99%^-^
PROBCONS 1.09	71.95%^-^	82.61%^-^	100.00%^-^	33.33%^0^	75.00%^-^	80.14%^-^
POA V2	20.73%^+^	34.78%^+^	16.67%^+^	33.33%^0^	0.00%^+^	21.99%^+^
MUSCLE 3.5	71.95%^-^	73.91%^-^	83.33%^-^	25.00%^0^	75.00%^-^	69.50%^-^
CLUSTAL W 1.83	53.66%^-^	56.52%^0^	58.33%^0^	16.67%^0^	8.33%^+^	47.52%^0^

**Table 8:** Percentage of sequence families where DIALIGN-T is outperformed on BAliBASE 2.1 by other methods according to the *column score*. Notation as in Table 5.

Method	ref1	ref2	ref3	ref4	ref5	Total
DIALIGN 2.2	26.83%^+^	13.04%^+^	16.67%^+^	16.67%^0^	50.00%^0^	24.82%^+^
T-COFFEE 1.37	56.10%^-^	73.91%^-^	66.67%^0^	25.00%^0^	50.00%^0^	56.74%^-^
PROBCONS 1.09	80.49%^-^	82.61%^-^	75.00%^-^	25.00%^0^	66.67%^-^	74.47%^-^
POA V2	20.73%^+^	26.09%^0^	08.33%^+^	16.67%^0^	00.00%^+^	18.44%^+^
MUSCLE 3.5	73.17%^-^	73.91%^-^	83.33%^-^	16.67%^0^	66.67%^-^	68.79%^-^
CLUSTAL W 1.83	52.44%^-^	69.57%^-^	50.00%^0^	16.67%^0^	08.33%^+^	48.23%^0^

**Table 9:** Average running time (in seconds) per multiple alignment for the 180 sequence families of IRMBASE and for 141 sequence families in BAliBASE 2.1. Program runs were performed on a Linux workstation (RedHat 8.0) with an 3.2 GHz Pentium 4 processor and 2 GB Ram.

Method	Average runtime on IRMBASE 1.0	Average runtime on BAliBASE 2.1
DIALIGN-T	2.36	1.38
DIALIGN 2.2	3.33	1.30
T-COFFEE 1.37	27.54	7.64
PROBCONS 1.09	12.37	2.66
POA V2	1.44	0.58
MUSCLE 3.5	9.37	0.60
CLUSTAL W 1.83	1.41	0.47
